# Fine-needle aspiration cytology of extra mammary metastatic lesions in the breast: A retrospective study of 36 cases diagnosed during 18 years

**DOI:** 10.4103/1742-6413.65056

**Published:** 2010-07-02

**Authors:** Torill Sauer

**Affiliations:** Address: Department of Pathology, Faculty Division of Clinical Medicine, Oslo University Hospital, Ulleval, N-0407 Oslo, Norway

**Keywords:** Breast, cytological features, extra mammary, FNAC, grade, metastases

## Abstract

**Background:**

Metastatic tumors in the breast require treatment according to origin and type of tumor. It is important to recognize these lesions in fine-needle aspiration cytology (FNAC) in order to avoid unnecessary mastectomy or non-relevant chemotherapy. The aim of this study was to evaluate the cytological features of metastatic tumors and possible criteria that could alert us as to the possibility of a metastasis from an extra mammary malignancy.

**Methods:**

The material included 36 confirmed or suspected metastases in the breast registered in the pathology files at Oslo University Hospital, Ulleval, during 1990–2007. There were a total of 6,325 cases of malignant breast FNAC, representing 30 men and 6,295 women. Smears were evaluated for the amount of material, presence or absence of myoepithelial cells, microcalcifications, mitoses and necrotic material. All carcinomas were graded.

**Results:**

There were seven men (7/30 = 23.3%) and 29 women (29/6,295 = 0.46%). The primary tumor was known in 22 cases (22/36 = 61.1%). No other primary tumor was known and metastatic lesion was not initially suspected in 14 cases (14/36 = 38.9%). The most common origin was lung (15/36 = 41.7%). In five cases (5/36 = 13.9%), the origin remained uncertain.

**Conclusions:**

Metastases from extra mammary sites are (relatively) common in males (23.3%). In women, metastatic lesions are rare (0.46%). A large proportion of them (88%) are high-grade adenocarcinomas and poorly differentiated carcinomas that may resemble grade 3 ductal carcinomas. Unusual clinical and/or radiological presentation in combination with high-grade malignant cells should alert us to consider the possibility of a metastasis.

## INTRODUCTION

Metastatic lesions in the breast are uncommon and make up about 3% of all tumors in the breast. Most of them are metastases from the contralateral breast and less than 0.5% will represent metastases from a primary tumor outside the breast.[1]

They are often located superficially, but cannot be differentiated from a primary breast tumor on clinical appearance. They often occur as palpable round tumors, firm and freely movable with no fixation of the overlying skin or the underlying pectoral muscle. There is no nipple discharge. Radiologically there may be a single or multiple nodules that tend to have the same size on palpation and mammography. Mammography typically show a circumscribed, round nodule with slightly irregular margins. There is usually no microcalcification or spiculation.

Microcalcification may occur, but usually as psammoma bodies in metastatic ovarian carcinomas as well as in metastatic lesions from the lung and the thyroid.[2–6]

Malignant lymphoma/leukemia and malignant melanomas are the most common non-epithelial metastases,[1,7–14] whereas lung, ovaries, kidney, thyroid, cervix, stomach, colon and prostate are the most common origins of epithelial malignancies.[1,8,12,13,15–21]

Metastatic tumors in the breast require treatment according to origin and type of tumor. Thus, it is important to recognize these lesions in FNAC in order to avoid unnecessary mastectomy or non-relevant chemotherapy. Many of the patients will have a previous history of a non-mammary malignancy, and thus, the cytopathologist may be alerted as to the possibility of a metastatic lesion. A variable number, though, up to 1/3 of the metastatic lesions may present themselves primarily in the breast without any other known primary.[1,13,19,20]

The aim of this study was to evaluate the cytological features of metastatic (non-hematological) tumors in the FNAC aspirates and extract possible criteria that could alert us as to the possibility of a metastasis from an extra mammary malignancy.

## MATERIALS AND METHODS

The material included all histological and/or cytological (FNAC) records of confirmed or suspected metastases in the breast (excluding from the contralateral breast) registered in the pathology files of Ullevaal University Hospital (UUS) during 1990–2007. All patients were dead. Their clinical records were closed and could not be accessed. Clinical information of an eventually known primary tumor, subtype and grading of tumor, relevant recent radiological findings other than in the breast were extracted from the pathology reports only, as well as results of immunohisto (IHC)- or cytochemistry (ICC), including estrogen (ER) and progesterone (PgR) receptor analysis. Cytological and/or histological material from the confirmed primary tumors was compared with the cytological findings from the breast lesions. FNAC had been done by a cytopathologist in all cases. Non-palpable lesions were aspirated under radiological guidance, either by ultrasonography or CT-guided, and together with a radiologist. Radiological and/or clinical information on the pathology records without cytology or histology from the probable origin was recorded as possible origin.

All ICC had been done on direct smears or preparations from liquid based suspensions. During this time period, there have been several organizational changes as to where the different disease groups in Oslo were treated and this is reflected in the spectrum of lesions in the breast referred for FNAC. Urological disorders and most of the gynecological oncology have been treated elsewhere for the whole period. The use of ICC has increased considerably since the earliest years and the in-house methods have been developing and changing over the years.

All smears were reexamined by the author and evaluated for a number of features. The amount of material (scant, moderate or abundant) as well as the presence or absence of myoepithelial cells, cytoplasmic vacuoles, microcalcifications, mitoses and necrotic material were recorded. Also, any cytological or histological materials from a known or possible primary tumor were compared with the tumor cells on the breast specimens. Grading of all carcinomas is done routinely on preoperative smears from breast carcinomas according to Robinson *et al*,[22] [Table 1] since five years. The evaluated criteria are dyscohesion, nuclear size, chromatin pattern, nucleoli and nuclear margin as well as cell uniformity. Grading was done and eventually repeated as part of this study.

**Table 1:** Cytological grading according to Robinson *et al*.[22]

*Criterion*	*Score 1*	*Score 2*	*Score 3*
Cell dissociation	Mostly clusters	Single cells and clusters	Mostly single cells
Nuclear size	1-2 times size of an erythrocyte	3-4 times size of an erythrocyte	≥ 5 times size of an erythrocyte
Cell uniformity	Monomorphic	Mildly pleomorphic	Pleomorphic
Nucleoli	Indistinct/ small	Noticeable	Abnormal
Nuclear margin	Smooth	Slightly irregular/folds and grooves	Buds and clefts
Chromatin pattern	Vesicular	Granular	Clumping and clearing

Score 6-11 = Grade 1, Score 12-14 = Grade 2m Score 15-18 = Grade 3

Histological material consisted of core needle biopsies, surgical specimens or autopsy material. All smears and histological material were reviewed by the author.

## RESULTS

During 1990-2007 6334 FNAC cases of malignant breast tumors were registered. There were 30 men and 6304 women. Malignant lymphoma or leukemia was diagnosed in nine women (primary or recurrence). These cases have been omitted from the present series. There were 36 cases of confirmed or suspected metastatic tumor in the breast, seven men (7/30 = 23.3%) and 29 women (29/6295 = 0.46%).

26/36 cases had either cytological or histological material from the primary site for comparison. All were found to be concordant with the findings in the breast on morphology and on ICC when that had been applied to both. In 10 cases there was no cytological or histological material from any other lesion. This is a limitation of the study, but most probably reflects that the patients have died before further investigations could be done.

An overview of origins and subtypes of the metastatic lesions is shown in Table 2. The primary tumor was known in advance in 17 cases (17/36 = 47.2%) [Table 3]. The suspected primary tumor and the breast metastasis were detected and investigated simultaneously in 5 cases (5/36 = 13.9%) [Table 3]. No other primary tumor was known and metastatic lesion was not initially suspected in 14 cases (14/36 = 38.9%) [Table 3]. In five of these (5/14 = 35.7%), a definite or possible origin of the tumor was not identified. The clinical records couldn’t be accessed for any information about these patients.

**Table 2:** Overview of origins and subtypes of metastatic tumors in the breast

*Origin*	*Subtype*	*Number of cases with cyt/hist confirmation*	*Number of cases with suspected origin but without cyt/hist confirmation*
Lung (15)	Adenocarcinoma	1	
	Small cell carcinoma	6 (2 males)	
	Poorly differentiated, non-small cell carcinoma	6	2 (1 male)
Stomach (4)	Adenocarcinoma	2	2 (1 male)
Colon (3)	Adenocarcinoma	3 (2 males)	
Ovaries (5)	Adenocarcinoma	3	2
Kidney (1)	Clear cell carcinoma	1	
Malignant melanoma (3)		3	
Soft tissue (1)	Malignant fibrous histiocytoma	1 (male)	

**Table 3:** Overview of whether the origin of the metastatic tumor was known or not

Primary tumor known (17/36 = 47.2%)
Lung carcinomas (= 6)
3 small cell carcinomas
1 adenocarcinoma
2 non-small cell carcinoma
3 ovarian carcinomas
3 colonic carcinomas (2 males)
1 MFH (male)
2 malignant melanomas
1 clear cell carcinoma of the kidney
2 adenocarcinomas from the stomach
Primary tumor and breast lesion detected and investigated simultaneously (5/36 = 13.9%)
2 small cell lung carcinomas (1 male)
3 non-small cell lung carcinomas
No (other) primary tumor known or suspected at the time of breast FNAC (14/36 = 38.9%)
1 malignant melanoma (incidentally detected at mammography screening)
1 small cell lung carcinoma (male)
1 non-small cell lung carcinoma (autopsy diagnosis 1 month after FNAC)
2 poorly differentiated carcinomas with a possible origin in the lungs (radiological infiltrate suspicious of malignancy), but not investigated further; no autopsy performed (1 male)
1 adenocarcinoma with suggested origin in the stomach (male) (but not confirmed before the patient died; no autopsy performed)
1 adenocarcinoma with suggested origin in pancreas or stomach (not investigated further; no autopsy performed)
2 adenocarcinomas probably originated in the ovaries (died before further investigation; no autopsy performed)
5 adenocarcinomas/poorly differentiated carcinomas with no specific clues as to origin; no autopsy performed

The most common origin was lung with 15 cases (15/36 = 41.7%). Of these, 12 cases were confirmed with a biopsy or cytology from the primary lesion. An additional case was diagnosed at autopsy one month after the investigation of the breast lesion. The lung was a possible or probable origin in another two cases that were not investigated further because the patients died. No autopsy was done.

The metastatic tumor originated in the ovaries in five cases (3 confirmed and 2 probable; 5/36 = 13.9%). Metastasis from malignant melanoma was found and confirmed in 3 cases (3/36 = 13.9%). The origin of the metastatic tumor was not definitely confirmed in 11 cases, but an origin was suggested according to clinical and/or radiological findings in six of them. In five cases (5/36 = 13.9%), no origin was confirmed or suggested [Table 3].

**Figure 1:**
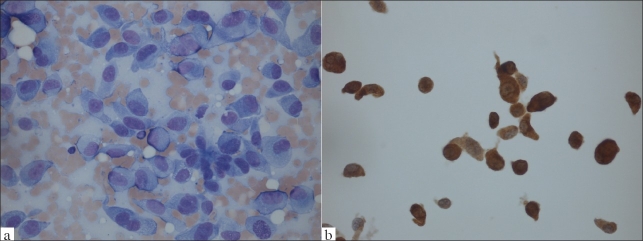
a) Malignant cells from metastatic malignant melanoma in the breast. Giemsa stain, Magnification ×400, b) Immunocytochemical staining with PanMel. Liquid based preparation, Magnification ×400

ER and PgR status on the cytological material was known in 20 cases (20/36 = 55.6%) and all were negative. ICC of other markers had been done in five cases. The results supported the cytological morphological diagnoses in a case of small cell carcinoma that was positive for TTF-1. The ICC was diagnostic in one case of metastatic malignant melanoma [Figures 1a‐b] and was non-contributory in a case where the origin remained uncertain and in 2 cases of probable ovarian carcinomas.

Grading was done on 32 carcinomas. Of these, six were small cell lung carcinomas and one was a clear cell renal carcinoma and the grading results were disregarded. The rest comprised 25 adenocarcinomas or poorly differentiated carcinomas without any specific subtype or differentiation and 22 of these (22/25 = 88%) were grade 3 [Table 4].

**Table 4:** Grading of adenocarcinomas and poorly differentiated carcinomas (n=25)

Grade 1(n=1)
1 adenocarcinoma
Grade 2 (n=2)
2 adenocarcinomas
Grade 3 (n=22)
17 adenocarcinomas
5 poorly differentiated carcinomas

The material was scant in eight cases (22.2%), moderate in nine cases (25%) and abundant in 19 cases (52.8%). Myoepithelial cells were not demonstrated in any of the specimens. Microcalcifications were found in five adenocarcinomas (from lung, colon, unknown and ovaries[2] and in the renal clear cell carcinoma. Necrotic debris was found in six adenocarcinomas, two poorly differentiated carcinomas and two small cell carcinomas. Cytoplasmic vacuoles were found in 14 adenocarcinomas and in one poorly differentiated carcinoma. Mitoses where identified in 8/38 cases, all of them adenocarcinomas (21%) with origin in colon,[2] ovaries,[2] lung, stomach and unknown.[2] None of these features (amount of material, presence or absence of myoepithelial cells, necrotic debris, cytoplasmic vacuoles and number of mitoses) were helpful in suspecting or diagnosing a metastatic lesion.

## DISCUSSION

Metastases from tumors outside the breast are rare. In our series they comprised 0.46% of the women which is the same as has been found by others.[1] This is in contrast to the findings in men, where 23.3% of the lesions were metastatic. When investigating male breast tumors, the possibility of a metastatic lesion should always be considered. The primary tumor was unknown in 38.9% of cases, which is also in accordance with previous findings.[1,13,19,20]

The histopathological appearance of primary breast carcinomas is very heterogeneous, and this is reflected in the FNAC specimens. We have a rather high tolerance level before we state that the morphology is unusual. Some of the extra mammary primaries will have a characteristic morphology, for example malignant melanoma,[7] most malignant lymphomas[11,14] and sarcomas as well as small cell carcinomas and clear cell carcinomas. They are usually readily recognized as “aliens”. If a preliminary stain is done, these lesions are recognized and additional material for ICC and eventually other molecular diagnostic methods can be obtained, either as direct smears or liquid suspensions. For the most part though, there were no specific cytological criteria in this material that would alert us to consider a metastatic lesion. The main pitfall in cases without any other known primary seems to be high grade and poorly differentiated carcinomas that resemble a high grade (G3) ductal carcinoma. In a large and busy FNAC clinic there will be several high grade carcinomas every week. Statistically there will be 2-3 metastatic lesions per year, and clinical and radiological findings are crucial to alert us to the possibility of a tumor being metastatic.

**Figure 2:**
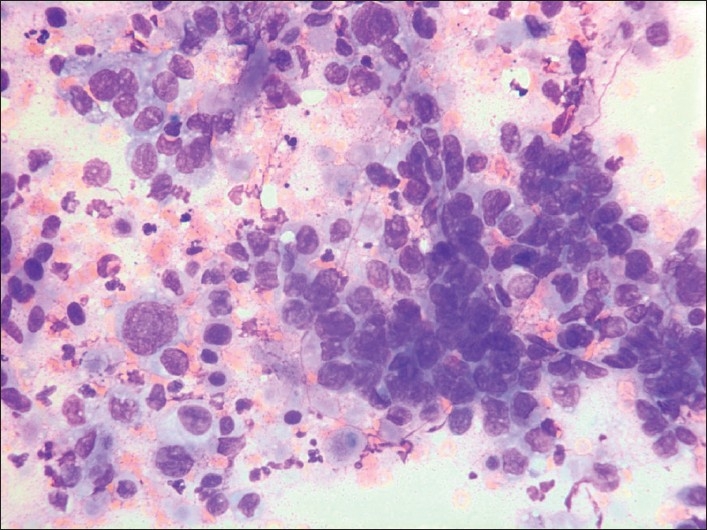
G3 cells from a primary lung carcinoma. Giemsa stain, Magnification ×400

**Figure 3:**
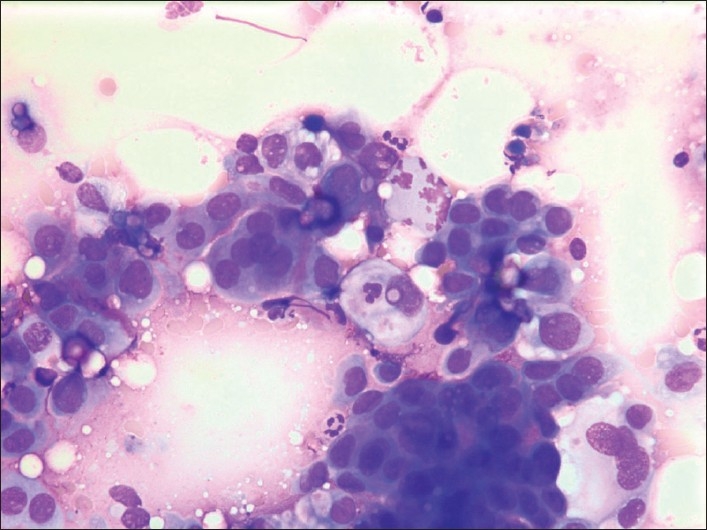
G3 cells from a primary ovarian carcinoma. Giemsa stain, Magnification ×400

**Figure 4:**
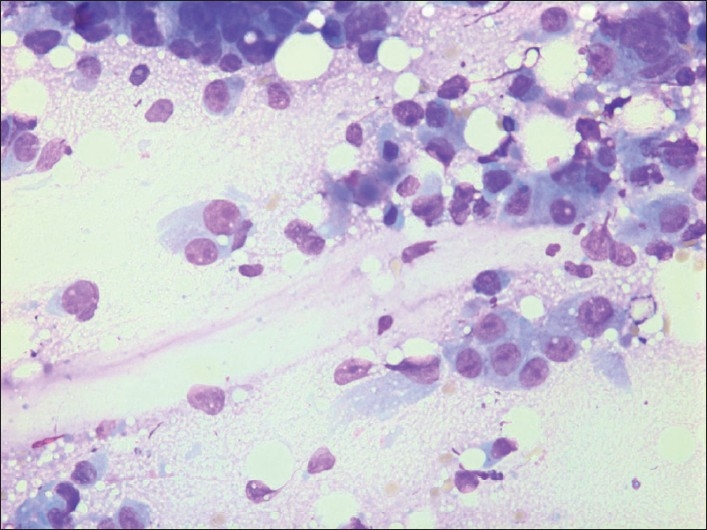
G3 cells from a primary carcinoma in the colon. Giemsa stain, Magnification ×400.

Grading does not seem to have been addressed in the context of metastases in breast FNAC previously. As such, it is not a discriminating feature in the diagnosis of a metastatic lesion, but most of the metastatic lesions were adenocarcinomas and/or poorly differentiated carcinomas with a high-grade nuclear and cellular atypia in our series where 22/25 (= 88%) adeno-/ poorly differentiated carcinomas were grade 3. They mainly originated in lung (41.7%) [Figure 2], ovaries [Figure 3], GI-tract [Figure 4] or had unknown origin. All of them may resemble high-grade ductal carcinoma and morphologically there are no features that specifically alert us. Additional features as psammoma bodies or papillary fragments, as may be found in ovarian and some lung and thyroid carcinomas, may be helpful.[15] If there is a previous (or simultaneous) history of extra mammary malignancy, additional material for ICC or other relevant diagnostic procedures should always be obtained. The antibody panel should include a cytokeratin panel and organ specific markers (as TTF-1, PSA, CDX2, pan-Mel, and LCA) depending on the previous primary. ER and PgR analysis may not always be contributory because a large number of high-grade ductal carcinomas are ER/PgR negative. Ovarian and endometrial carcinomas may well be ER/PgR positive as well. Occasionally, ER/PgR positivity may be found in cells and lesions from other organs such as lung and liver. TTF-1 positivity is a good indicator (but not a proof) of a primary lung carcinoma,[23] but up to 25%[24,25] of the adenocarcinomas are negative and most metastatic thyroid carcinomas would be positive. ICC had limited impact on the diagnoses in our series. In most cases no or few additional smears or liquid suspensions were available for ICC. This is mainly due to lack of opportunity to do ICC in the earliest years. However, in later years, the main reason was probably that the aspirator(s) had not initially suspected a metastatic lesion. The critical cases are those who do not have a previous or simultaneous history of malignancy and where FNAC reveal cells from a high-grade carcinoma. In our series, 5 cases in this group remained unresolved as to origin of the tumor [Table 3]. Today, more extensive use of ICC might have allowed all or most of them a more specific diagnosis as to origin.

In conclusion, metastases from extra mammary sites are relatively more common in males (23.3%) than in women and should always be a differential diagnosis in the work-up of breast tumors in men. In women, metastatic lesions are rare (0.46%). A large proportion of the metastases (88%) are high-grade adenocarcinomas and poorly differentiated carcinomas that may resemble grade 3 ductal carcinomas. That means that a large majority of metastatic tumors to the breast have no specific features. Unusual clinical and/or radiological presentation in combination with cell material from a high-grade/poorly differentiated carcinoma should alert us to consider the possibility of a metastasis, and thus, the need for immunocytochemical investigation. A complete knowledge of the patient history is crucial.

## COMPETING INTEREST STATEMENT BY ALL AUTHORS

No competing interest to declare by any of the authors.

## AUTHORSHIP STATEMENT BY ALL AUTHORS

Each author acknowledges that this final version was read and approved. All authors of this article declare that we qualify for authorship as defined by ICMJE http://www.icmje.org/#author. Each author has participated sufficiently in the work and take public responsibility for appropriate portions of the content of this article.

## ETHICS STATEMENT BY ALL AUTHORS

This study was conducted with approval from Institutional Review Board (IRB) (or its equivalent) of all the institutions associated with this study. Authors take responsibility to maintain relevant documentation in this respect.

## EDITORIAL / PEER-REVIEW STATEMENT

To ensure integrity and highest quality of CytoJournal publications, the review process of this manuscript was conducted under a double blind model(authors are blinded for reviewers and reviewers are blinded for authors) through automatic online system.
